# Gene Edited T Cell Therapies for Inborn Errors of Immunity

**DOI:** 10.3389/fgeed.2022.899294

**Published:** 2022-06-16

**Authors:** T. A. Fox, B. C. Houghton, C. Booth

**Affiliations:** ^1^ UCL Institute of Immunity and Transplantation, University College London, London, United Kingdom; ^2^ Department of Clinical Haematology, University College London Hospitals NHS Foundation Trust, London, United Kingdom; ^3^ Molecular and Cellular Immunology Section, UCL GOS Institute of Child Health, London, United Kingdom; ^4^ Department of Paediatric Immunology, Great Ormond Street Hospital for Sick Children NHS Foundation Trust, London, United Kingdom

**Keywords:** primary immumunodeficiencies, inborn error of immunity (IEI), gene editing, t cell, cellular therapeutics, gene therapeutics

## Abstract

Inborn errors of immunity (IEIs) are a heterogeneous group of inherited disorders of the immune system. Many IEIs have a severe clinical phenotype that results in progressive morbidity and premature mortality. Over 450 IEIs have been described and the incidence of all IEIs is 1/1,000–10,000 people. Current treatment options are unsatisfactory for many IEIs. Allogeneic haematopoietic stem cell transplantation (alloHSCT) is curative but requires the availability of a suitable donor and carries a risk of graft failure, graft rejection and graft-versus-host disease (GvHD). Autologous gene therapy (GT) offers a cure whilst abrogating the immunological complications of alloHSCT. Gene editing (GE) technologies allow the precise modification of an organisms’ DNA at a base-pair level. In the context of genetic disease, this enables correction of genetic defects whilst preserving the endogenous gene control machinery. Gene editing technologies have the potential to transform the treatment landscape of IEIs. In contrast to gene addition techniques, gene editing using the CRISPR system repairs or replaces the mutation in the DNA. Many IEIs are limited to the lymphoid compartment and may be amenable to T cell correction alone (rather than haematopoietic stem cells). T cell Gene editing has the advantages of higher editing efficiencies, reduced risk of deleterious off-target edits in terminally differentiated cells and less toxic conditioning required for engraftment of lymphocytes. Although most T cells lack the self-renewing property of HSCs, a population of T cells, the T stem cell memory compartment has long-term multipotent and self-renewal capacity. Gene edited T cell therapies for IEIs are currently in development and may offer a less-toxic curative therapy to patients affected by certain IEIs. In this review, we discuss the history of T cell gene therapy, developments in T cell gene editing cellular therapies before detailing exciting pre-clinical studies that demonstrate gene editing T cell therapies as a proof-of-concept for several IEIs.

## 1 Introduction

Inborn errors of Immunity (IEIs) (also known as primary immunodeficiencies) are a heterogeneous group of rare inherited disorders of the innate or adaptive immune system (Notarangelo, 2010; Picard et al., 2018). There are over 400 distinct IEIs and new diseases are regularly described as our understanding of genetic defects and immune phenotypes increase (Parvaneh et al., 2013). Individually IEIs are rare disorders. The prevalence of IEIs collectively is estimated to be 50 patients per 100,000 population (Kobrynski et al., 2014).

IEIs have heterogeneous clinical phenotypes within phenotypic groups and even within individual diagnoses. Recurrent infections (often severe, life-threatening infections) are the most common presenting complaint. An underlying IEI should be suspected in patients presenting with a wide variety of haematological and immunological symptoms at an earlier than expected age, which are refractory to conventional treatments or who have a relevant family history (Fox and Booth, 2021). For severe combined immune deficiencies (SCID), survival past infancy is rare without a definitive treatment (Cirillo et al., 2019). For non-SCID IEIs, clinical severity is variable but frequently result in progressive morbidity and premature mortality (Pai, 2019).

Allogeneic haematopoietic stem cell transplantation (alloHSCT) is the only curative treatment for most IEIs. AlloHSCT is performed as standard of care for SCID. An increasing number of patients with non-SCID IEIs who have developed a severe manifestation of their disease, usually considered to be serious or life-threatening infections, refractory autoimmunity, malignancy, or the development of multi-organ damage as a complication of their disease are considered for alloHSCT (Pai and Cowan, 2014; Fox et al., 2018; Burns and Morris, 2021). Whilst curative, alloHSCT requires a suitable donor and carries a risk of graft failure, graft rejection, and graft versus host disease (GvHD) (Fox et al., 2018; Burns and Morris, 2021).

Autologous *ex vivo* haematopoietic stem cell gene therapy (HSC-GT) is in the advanced stages of clinical development for several IEIs. HSC-GT may potentially offer curative therapy whilst mitigating the immunological complications of alloHSCT (Booth et al., 2016; Fox and Booth, 2021). The development of gene therapy using retroviral vectors and lentiviral vectors to transduce HSCs has had a turbulent history, which has been summarised in several recent reviews (Booth et al., 2016; Kuo and Kohn, 2016; Kohn and Kuo, 2017; Fox and Booth, 2021). Early viral vectors resulted in insertional mutagenesis in several patients after transgene integration occurred close to proto-oncogenes leading to leukaemias and myelodysplasia in several patients in trials (Howe et al., 2008; Braun et al., 2014; Dunbar et al., 2018; Fox and Booth, 2021).

In response to the adverse events seen in retrovirus vector GT trials, the field focused on developing new vectors with an improved safety profile. Retroviral vectors use powerful viral promoters in the long terminal repeat (LTR) sequences to drive transgene expression and have a preference for integration in transcriptionally active genomic regions (Scherdin et al., 1990; Fox and Booth, 2021). The development of self-inactivating (SIN)-retroviral-vectors incorporated mutations in the LTRs and less powerful mammalian promoters (Gaspar et al., 2011; Hacein-Bey-Abina et al., 2014). At the same time a novel vector based on the human immunodeficiency virus (HIV), a lentivirus, was developed that has removed the pathogenic potential of the virus with an integration pattern associated with a lower risk of oncogene activating insertions (Modlich et al., 2009; Biffi et al., 2011). Additional advantages of lentiviral vectors over retroviral vectors are improved efficiency of gene transfer and the ability to transduce both proliferating and non-proliferating cells, reducing the amount of *ex vivo* manipulation required to produce a HSC-GT product (Sutton et al., 1999; Millington et al., 2009; Modlich et al., 2009; Montini et al., 2009).

The development of self-inactivating lentiviral vectors has resulted in several promising and safe HSC-GT approaches for several IEIs including different forms of SCID, chronic granulomatous disease (CGD), and Wiskott-Aldrich syndrome (Ferrua et al., 2019a; Kohn et al., 2020; Kohn et al., 2021).

**FIGURE 1:**
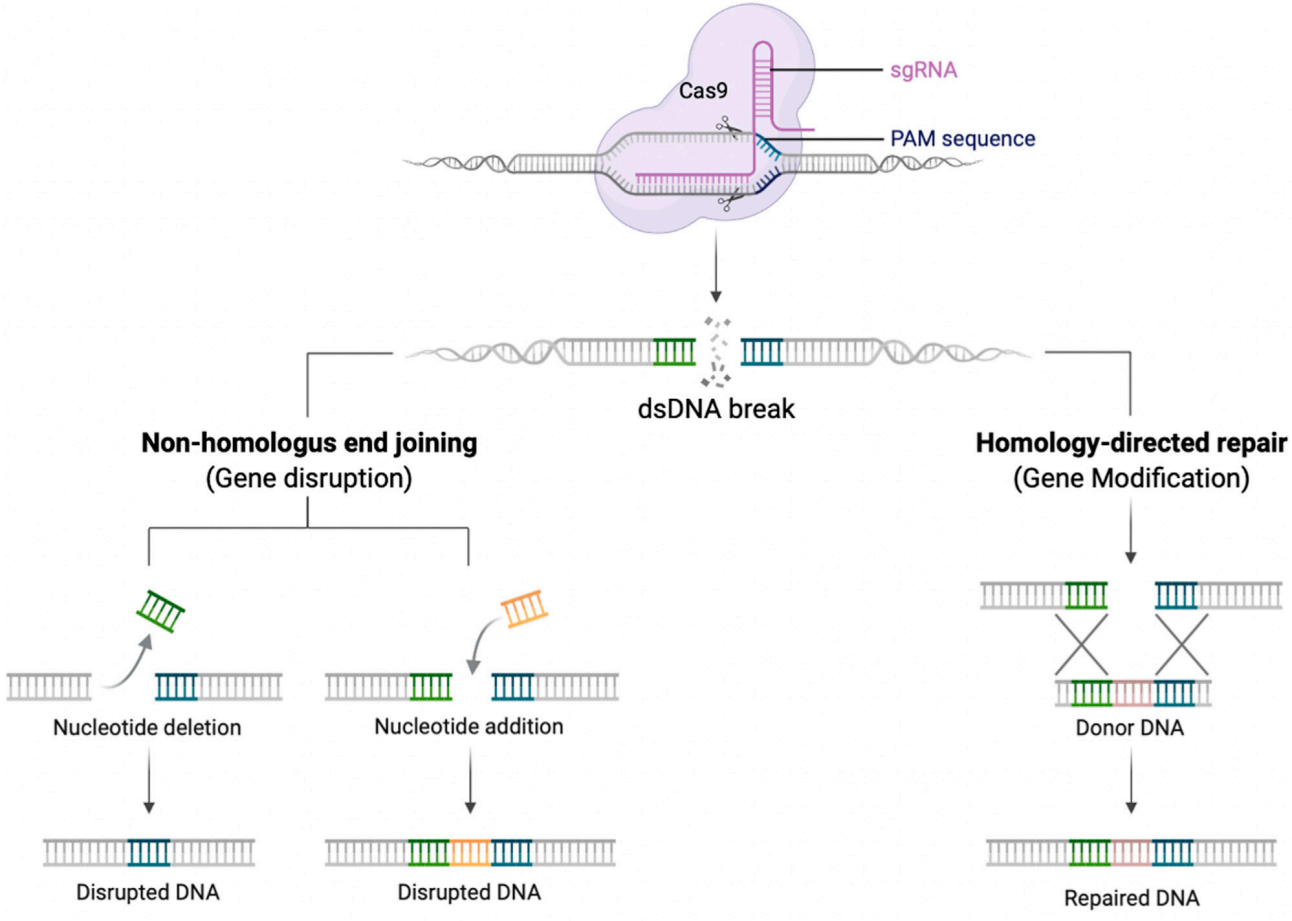
Schematic diagram demonstrating the gRNA/Cas9 complex performing a dsDNA break. Once the dsDNA break is created, the DNA repairs itself by NHEJ creating insertions and deletions (left pathway), or in the presence of a donor DNA template HDR can occur with insertion of new genetic material (right pathway). Created with biorender.com.

It is challenging to replicate physiological gene expression profiles using lentiviral vectors, and their application to some disorders is constrained by size limitations of expression cassettes (Porteus, 2019). Gene editing (GE) technologies allow the precise modification of an organisms’ DNA at a base-pair level. In the context of genetic disease, this enables correction of genetic defects whilst preserving the endogenous gene control machinery (Porteus, 2019). Several designer DNA endonucleases are available that can introduce a double-stranded DNA break at a specific target sequence, including Transcription activator-like effector nuclease (TALENs), zinc-finger nucleases (ZFNs), and engineered meganucleases. More recently, the CRISPR-Cas9 (clustered regularly interspaced short palindromic repeats associated with Cas9 endonuclease) RNA-based system has further spurred the development of gene editing strategies due to its comparative ease of use (Jinek et al., 2012) (Figure 1).

Once a double strand break is created, DNA will preferentially repair by the process of non-homologous end joining (NHEJ). This error prone mechanism, results in insertions and deletions (indels) at the site of the dsDNA break. NHEJ can occur during all phases of the cell cycle and be exploited to knock-out a gene (Naldini, 2019). In contrast, if an exogeneous DNA repair template is provided, that contains sequences homologous to the 5′ and 3′ of dsDNA break, repair can occur by the process of homology directed repair (HDR). This process is restricted to cells in S/G2 phases of the cell cycle but is highly precise and permits the seamless insertion of new genetic material (Bak et al., 2018; Naldini, 2019).

### 1.1 A New Class of Gene Therapy—T Cell Gene Therapy

Adoptive transfer of gene-modified T cells has been used for over 20 years as an experimental treatment strategy for a variety of disorders most notably in haematological malignancies, with chimeric antigen receptor T (CAR-T) cell therapy. T cell adoptive cellular therapy has a strong safety profile with over 500 years of patient follow-up (Scholler et al., 2012). Several IEIs arise predominantly due to defects within the T cell compartment, thus correction of T cell populations alone may ameliorate clinical manifestations in conditions such as CD40 ligand deficiency, Immune dysregulation polyendocrinopathy enteropathy X-linked (IPEX) syndrome, X-linked lymphoproliferative syndrome (XLP), cytotoxic T-lymphocyte antigen 4 (CTLA4) insufficiency and signal transducer and activation of transcription 1 (STAT1) loss of function/gain of function mutations.

T cell GT is proposed as a novel approach to correct genetic disorders that has several advantages over HSC modification (Table 1). Firstly, large numbers of T-lymphocytes can be obtained with non-mobilised apheresis. The lymphodepletion required prior to infusion of a T-cell product is significantly less toxic than the myeloablative regimens required for HSC engraftment (Jain et al., 2019; Bechman and Maher, 2021). Cyclophosphamide is one of the most effective lymphodepleting agents and is usually combined with fludarabine for the purposes of pre-conditioning prior to infusion of T cell adoptive cellular therapy (Amini et al., 2022). As T-cells are terminally differentiated, the risk of insertional mutagenesis is reduced, although the long term follow up will be required to detect any late genotoxic events (Scholler et al., 2012). Long-term follow up is as important in T cell GT as HSC-GT as modification of T stem cell memory cells could potentially enable the genetically modified cells to persist and proliferate for the lifetime of the patient. *In vitro* proof of concept studies have now been published for T cell GT using lentiviral vector gene addition for XLP, CD40 ligand deficiency, IPEX, perforin deficiency and MUNC13-4 deficiency [the latter two are forms of familial haemophagocytic lymphohistiocytosis (HLH)] (Passerini et al., 2013; Hubbard et al., 2016; Soheili et al., 2016; Ghosh et al., 2018; Kohn et al., 2021). The disadvantages of T cell GT compared to HSC GT are the lack of correction across all haematopoietic lineages (limiting the application to disorders predominantly mediated through the lymphoid compartment and the potentially shorter-term persistence of gene-corrected T cells. It is not yet known what degree of correction/chimerism is needed to ameliorate the clinical phenotype in T cell mediated IEIs. This is likely to be disease dependent and would need to be assessed in clinical trials. Data from studies of alloHSCT could be used to estimate the degree of correction required. For example, in patients who had undergone alloHSCT for CD40 ligand deficiency, in survivors who were able to cease immunoglobulin replacement therapy, T lymphocyte chimerism was over 50% donor (Ferrua et al., 2019b).

**TABLE 1:** Advantages and disadvantages of alloHSCT compared to HSC GT and T cell GT.

	AlloHSCT	HSC GT (viral vector or gene editing)	T Cell GT (viral vector or gene editing)
Advantages	• Widely available	• Promising clinical efficacy across a range of IEIs	• Lymphocytes can be collected from non-mobilised apheresis
	• Corrects all haematopoietic lineages	• Autologous, no need for donor, no risk of GvHD	• Only lymphodepletion required prior to infusion (less toxic)
	• Proven efficacy across a range of IEIs	• Corrects all haematopoietic lineages	• Autologous, no need for donor, no risk of GvHD
			• Less risk of insertional mutagenesis in terminally differentiated cells
Disadvantages	• Requires availability of a suitable donor	• Risk of insertional mutagenesis	• Limited to IEIs confined to lymphoid compartment
	• Risk of GvHD	• Requires conditioning that depletes HSCs (e.g., busulphan/melphalan)	• Experimental, efficacy of T cell GT for IEIs remains to be determined
	• Risk of graft failure/graft rejection	• Requires mobilised apheresis to harvest HSCs	• Persistence may be an issue
	• Requires conditioning that depletes HSCs (e.g., busulphan/melphalan)		
	• Donor needs to undergo mobilised apheresis		

In this review, we will describe the history and development of T cell cellular therapies and GT. We will outline the application of GE to T cell GT and describe the proof-of-concept studies that have used GE to correct IEIs in T cells. We will look to the future and describe the developments currently underway which may permit *in vivo* T cell GT for IEIs before outlining the techniques and potential issues associated with manufacturing a GE T cell GT product.

### 1.2 History of T Cell Gene Therapy

The use of genetically modified T cells for therapeutic purposes began in the 1980s with clinical trials of virus-specific T cells and tumour infiltrating lymphocytes (Kradin et al., 1989; Panchal et al., 2021). However, it wasn’t until the dramatic clinical results seen with chimeric antigen receptor T (CAR-T) cell therapy in B cell haematological malignancies that genetically modified T cell immunotherapy entered mainstream clinical practice (Maude et al., 2014; Maude et al., 2015; Brown and Shah, 2018).

T cell gene therapy in IEIs predates CAR-T therapy. In the 1990s, several groups modified T cells from patients with adenosine deaminase deficient (ADA) SCID using gammaretroviral vectors (Blaese et al., 1995; Bordignon et al., 1995). ADA-SCID presents in early infancy with life-threatening infectious complications and was universally fatal prior to alloHSCT for the condition in the 1960s (Blaese et al., 1995; Bordignon et al., 1995; Sauer and Aiuti, 2009; Dvorak et al., 2014; Kohn et al., 2019). In most of the patients treated on these early clinical trials of T cell GT for ADA-SCID, T cell function was restored following infusion of the gene corrected T cells (Blaese et al., 1995; Bordignon et al., 1995). Much was learnt about the therapeutic potential of T cell GT from these early trials. Gene modified T cells were demonstrated to have a similar cytokine release profile following stimulation. Patients developed a polyclonal T cell receptor repertoire after infusion of the modified T cells (Blaese et al., 1995; Bordignon et al., 1995; Kohn et al., 1995; Kohn et al., 1998).

Remarkably, in the patients which did not receive another definitive treatment such as alloHSCT, the genetically modified T cells were still detectable in patients >10 years post GT. Although genetically corrected T cells in ADA-SCID would have a survival advantage over uncorrected cells, central memory CD8^+^ T cells (CD8^+^ CD45RA + CD62L+) and T stem cell memory cells (CD45RA + CD62L−CD95^+^) were found to be enriched relative to other T cell subsets in patients with prolonged persistence of genetically modified T cells (Blaese et al., 1995; Bordignon et al., 1995; Kohn et al., 1995). It is now well established that these populations of cells are necessary for T cell persistence, and the detection of these cells in patients post T cell GT suggests that T cell GT may provide long term therapeutic benefits in patients with certain conditions (Biasco et al., 2015; Panchal et al., 2021). Long term persistence of genetically modified T cells has been seen with chimeric antigen receptor T (CAR-T) cells for the treatment of haematological malignancies. Recent data has shown over decade-long remissions with a detectable, stable, gene-modified CD4^+^ T cell population (Melenhorst et al., 2022).

Despite the T cell correction and persistence, ADA-SCID results in absence of B and NK cells (in addition to T cells) thus, ultimately HSC-GT has been developed for the condition in order to achieve higher levels of ADA via multi-lineage immune reconstitution (Aiuti et al., 2002; Ferrua and Aiuti, 2017; Kohn et al., 2021). Apart from these early trials in ADA-SCID, there is no human clinical trial data to support a T cell approach in IEIs or other inherited conditions. There have been several promising pre-clinical proof-of-concept studies using viral vector gene addition and gene edited T cell GT approaches published and several of these are expected to enter clinical trials in the near future. These studies along with data on T cell gene editing for adoptive cellular therapy will be discussed subsequently as they provide a strong rationale for the development of T cell GT, particularly using gene editing to correct the cellular defect.

### 1.3 Gene Addition T Cell Gene Therapy for Inborn Errors of Immunity

Although the focus of this review is on gene edited T cell therapies for IEIs, it is pertinent to consider T cell GT strategies in development that use viral-mediated gene addition to genetically modify cells. As viral vector mediated gene addition techniques have been in development for longer than gene editing techniques, therapeutic approaches using this technique are at more advanced stages of development and are likely to enter human clinical trials before gene edited T cell GT. The first clinical trials of T cell GT in a non-SCID IEI will be pivotal in assessing the viability of this strategy. Although the trials of T cell GT for ADA-SCID were promising in terms of T cell correction, function, and persistence, this was not an ideal disease to apply T cell GT to, due to the requirement for high levels of systemic secretion of ADA, that was unlikely to be met by a partially corrected T cell compartment. As a result, the IEIs currently being considered for a T cell approach are predominantly due to T cell defects.

#### 1.3.1 X-Linked Lymphoproliferative Disease

X-linked lymphoproliferative disease type 1 is caused by mutations in the *SH2D1A* (XLP1) gene which encodes the signalling lymphocytic activation molecule (SLAM)-associated protein (SAP). SAP is an intracellular adaptor protein expressed in T, NK, and NKT cells. In the presence of SAP, SLAM functions as an activating receptor whereas in the absence of SAP, it is inhibitory (Panchal et al., 2018a; Xu et al., 2020). Clinically, the disorder is characterized by severe immune dysregulation and complications often triggered by EBV infection, such as hemophagocytic lymphohistiocytosis (HLH), lymphoproliferation and colitis (Booth et al., 2011; Panchal et al., 2018a; Xu et al., 2020). Humoral defects result from T follicular helper cell (T_FH_) dysfunction, and include hypogammaglobulinaemia and impaired responses to vaccination (Booth et al., 2011; Panchal et al., 2018a). AlloHSCT is currently the only curative therapy, however a suitable donor is required and outcomes depend on whether adequate disease control can be achieved prior to transplant (Booth et al., 2011). We proposed that alloHSCT should be performed in all children diagnosed with XLP1, since once the patients develop HLH, they have a significantly reduced survival (Booth et al., 2011).

Proof-of-concept HSC-GT for XLP was first established by Rivat et al. (2013), who tested an HSC-GT approach using a lentiviral vector containing the codon-optimised SAP cDNA driven by an endogenous human elongation factor 1 alpha (EFS) promoter. Restoration of CD8^+^ T cell and NK cell cytotoxicity was observed in a SAP^−/γ^ murine model following adoptive transfer of haematopoietic progenitors transduced with the lentiviral vector (Rivat et al., 2013). Although no adverse consequences of vector expression in HSCs were observed in this study, SAP is normally tightly regulated and is not normally expressed in HSCs. The T cell defect that is predominant in XLP could thus be addressed through a T cell GT strategy which would avoid non-physiological SAP expression in other haematopoietic lineages. Proof-of-concept for T cell GT was established by Panchal et al. (2018b) who demonstrated *in vitro* correction of patient cells following transduction with a lentiviral vector. The authors went on to demonstrate that correction of humoral and cytotoxic defects could be achieved by transfer of gene-corrected T cells in an *in vivo* murine model of the disease (Panchal et al., 2018b). This approach is expected to enter a human clinical trial in the near future.

#### 1.3.2 Immune Dysregulation, Polyendocrinopathy, Enteropathy, X-Linked Syndrome

Defects in the transcription factor FOXP3 which is required for the normal development and function of T regulatory cells (Tregs) results in IPEX syndrome and the clinical manifestations of enteropathy, type I diabetes mellitus and eczema. Long-term immunosuppression can be used for disease control and alloHSCT is potentially curative but can be affected by immune-mediated complications (Barzaghi et al., 2018).

It has been demonstrated that adoptive transfer of wild type CD4^+^ CD25^+^ (WT) Tregs controlled the disease phenotype in a FOXP3^mut^ (scurfy) murine model of IPEX syndrome. Passerini *et al*, developed a lentiviral vector that results in constitutive FOXP3 expression, driven by an EF1a promoter. Transduction of IPEX conventional T cells with this vector converted them to functional suppressive Tregs (Passerini et al., 2013). It is unknown whether these Tregs would persist or whether they could generate unwanted immunosuppression *in vivo*. Successful lentiviral vector modification of HSCs for IPEX syndrome has also been performed and both murine and humanised mouse models have been used to demonstrate that these transduced HSCs are able to differentiate to functional Tregs *in vivo* (Masiuk et al., 2019). The efficacy of T cell versus HSC GT for IPEX syndrome would need to be determined in a clinical trial.

#### 1.3.3 Primary (Familial) Hemophagocytic Lymphohistiocytosis

Primary Hemophagocytic Lymphohistiocytosis (HLH) is a rare, life-threatening disorder characterized by hyperinflammation and uncontrolled immune activation that classically presents with a triad of splenomegaly, fever and cytopenias (Parikh et al., 2014). Primary HLH can be caused by genetic mutations primarily affecting Natural Killer (NK) and cytotoxic T cell function. These include familial HLH caused by autosomal recessive mutations in Perforin (*PRF1*), MUNC 13-4 (*UNC13D*), MUNC 19-2 (*STXBP2*), syntaxin 11 (*STX11*) as well as other IEIs such as Type II Hermansky-Pudlak syndrome (*AP3B1*), Chediak-Higashi syndrome (*LYST*) and Griscelli syndrome (*RAB27A*) (Hayden et al., 2016). All of these are classified as immune dysregulation disorders.

Primary HLH most commonly presents in childhood however, whilst the incidence in adults is not known, there are increasing reports in the literature describing patients aged over 18 at initial presentation (Schram and Berliner, 2015). Presentation of primary HLH can be dramatic and life-threatening and combinations of chemotherapy and immune suppression including corticosteroids, targeted biologics [e.g., anti-interferon gamma (IFNγ) monoclonal antibodies] are required to bring the inflammation under control. Once initial disease control is achieved, patients with a detectable genetic mutation, or refractory or relapsed primary HLH in the absence of a genetic diagnosis undergo alloHSCT if they are able (Marsh et al., 2010; Schram and Berliner, 2015).

T cell GT is under investigation for several forms of primary HLH to act either as a bridge to definitive therapy (HSC GT or alloHSCT) or, depending on the ability of the modified T cells to persist *in vivo*, as a potential curative therapy. Mutations in perforin are the commonest cause of familial HLH (accounting for 58% of cases) and it has been previously shown that adoptive transfer of functional WT T cells can protect against episodes of HLH in a murine model of the disease (Terrell and Jordan, 2013). Ghosh *et al* devised a gammaretroviral vector and demonstrated that it could be used to transduce murine *prf* ^*−/−*^ CD8^+^ T cells. Adoptive transfer of these corrected T cells into prf^−/−^ mice resulted in functional correction with reduced interferon-γ levels in mice who received corrected cells compared to negative controls (Ghosh et al., 2018). The authors then designed a similar, clinically applicable, lentiviral vector and demonstrated that transduction of peripheral blood mononuclear cells (PBMCs) from a patient with perforin deficiency resulted in restoration of cytotoxic function (Ghosh et al., 2018).

Several groups are working on gene addition T cell (and HSC) GT strategies for familial HLH which results from mutations in the *UNC13D* gene (encoding MUNC 13-4 protein) which accounts for 30%–40% of FHL cases. Both retroviral and lentiviral constructs have been developed and, following transduction of CD8^+^ T cells, have restored cytotoxicity both *in vitro* and *in vivo* (Soheili et al., 2016; Soheili et al., 2017; Dettmer et al., 2019).

A major challenge to autologous GT (both T cell and HSC) in familial HLH syndromes, however, is the difficulty collecting autologous T cells or stem cells in patients with systemic severe autoinflammation. If and once control of HLH is achieved, the window to deliver a definitive curative therapy may be short and, provided a suitable donor is available, alloHSCT may be more practical than manufacturing a GT product. Whilst T cell GT is a promising approach, the role of GT in the treatment pathway is not yet clear and careful thought will need to be given to the design of a GT trial in familial HLH.

### 1.4 T Cell Gene Editing for Adoptive Cellular Therapy

Before considering T cell gene editing for IEI GT, it is important to consider how gene editing is beginning to enter the field of T cell adoptive cellular therapy. T cell adoptive cellular therapy refers to the administration of T cells to a patient to fight cancer. It includes genetically modified T cells, clonally expanded virus-specific T cells, chimeric antigen receptor T cells (CAR-T) and T cell receptor (TCR) transduced T cells. Gene editing has been applied to modify and enhance T cell function and several gene edited T cell therapies have entered human clinical trials (Gill et al., 2016; Houghtelin and Bollard, 2017; Chapuis et al., 2019).

CAR-T cells have generated much excitement since early human clinical trials in B-cell acute lymphoblastic leukaemia demonstrated high rates of complete remission in patients who were refractory to all other available therapies (Porter et al., 2011; Lee et al., 2015). CAR-Ts are generated by transducing T cells with retroviral or lentiviral vectors expressing the CAR construct. However, it has been shown that editing a T cell with CRISPR/Cas9 and AAV HDR template to the T-cell receptor *a* constant (TRAC) locus enhanced CAR-T cell potency and edited cells outperformed CAR-T cells generated using viral transduction methods (Eyquem et al., 2017). This approach is currently being assessed in a clinical trial (NCT04035434).

Most CAR-T cells to date use autologous T cells, however in heavily pre-treated patients, particularly infants, it can be difficult to obtain enough lymphocytes to manufacture an effective therapeutic product. Gene editing is thus being used to generate ‘off-the-shelf’ allogenic CAR-T cells and the first-in-human application of this strategy was performed using TALENs to edit the TRAC and CD52 gene loci with successful results (Qasim et al., 2017; Qasim et al., 2018). Several trials of allogeneic CAR-T cells that have undergone gene editing to disrupt T cell receptor expression or other pathways are underway (NCT04557436, NCT04637763). Although allogeneic CAR-T cells appear to be as effective as autologous cells at tumour killing, autologous cells have the advantage of potential long-term persistence which may be needed for durable remissions (Depil et al., 2020). Work aimed at improving the persistence of allogeneic CAR-T cells includes using gene editing to delete MHC class I molecules by disrupting β2-microglobulin (to prevent immune rejection) and/or make them resistant to alemtuzumab by disrupting CD52 (Wang et al., 2015; Qasim et al., 2018). Several of these approaches are currently being assessed in clinical trials (NCT04502446, NCT04637763).

T cell receptor (TCR) transduced T cells comprise an engineered alpha (*α*) beta (*β*) heterodimer with endogenous signalling molecules (Chapuis et al., 2019). TCR transduced cells recognise peptide in the context of MHC and have the ability to be stimulated by single antigen molecules, which is achieved by serial low affinity TCR: peptide-MHC interactions (Stauss and Tran, 2020). Although the efficacy seen with CD19 CAR-T cells has not been demonstrated in humans with TCR-engineered T cells, the TCR approach has a significant advantage over CAR-T technology in that intracellular antigens can be targeted (Stauss and Tran, 2020). Recent data has shown that TCR transduced T cells targeting the Wilms’ Tumour antigen 1, are able to prevent AML relapse post-transplant (Chapuis et al., 2019). One of the potential disadvantages of TCR-engineered T cells is the risk of mispairing between the *α* and *β* chains of the introduced TCR and endogenous TCRs, potentially creating a new TCR with unknown off-target specificity. Gene editing has been used to mitigate this risk by disrupting the endogenous TCR *α* and *β* chains (Provasi et al., 2012; Stadtmauer et al., 2020).

### 1.5 Proof-of-Concept Studies of Gene Editing T Cell Gene Therapy for Inborn Errors of Immunity

**FIGURE 2:**
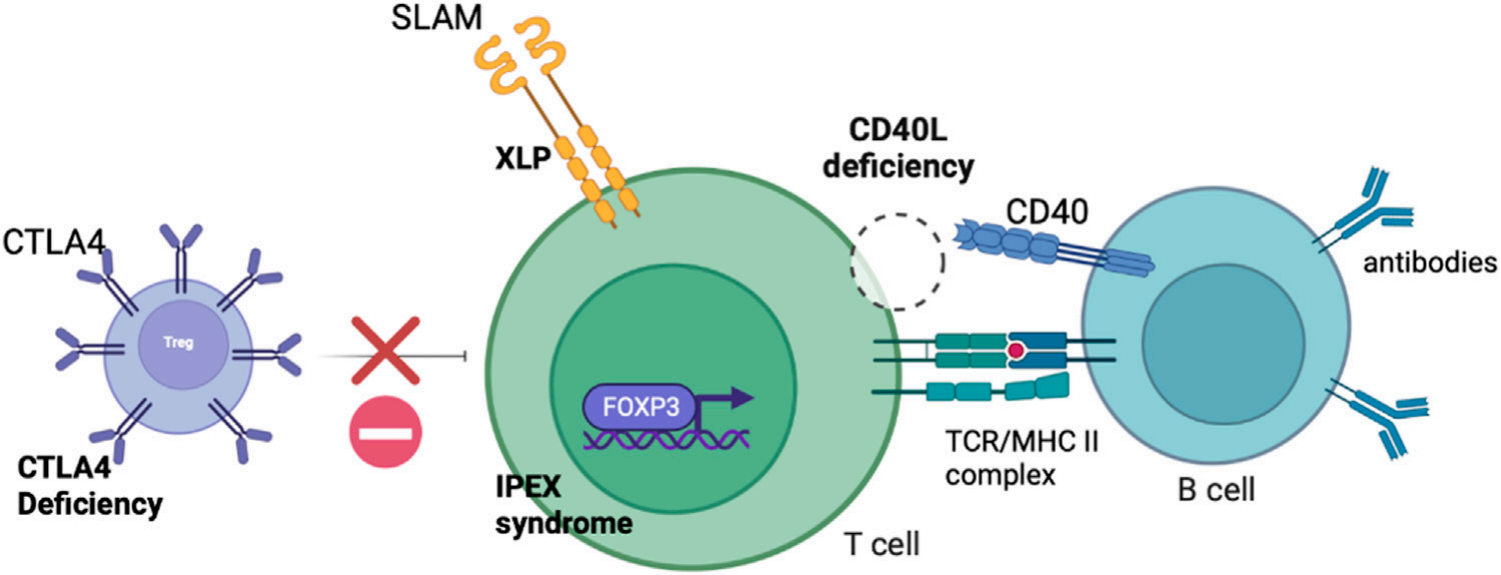
Schematic diagram demonstrating the defects present in IEIs with predominant lymphocyte defects potentially amenable to correction with T cell gene editing.

IEIs where the defect is predominantly confined to the lymphoid compartment may be corrected with T cell gene edited GT. As described, gene addition T cell GT is likely to enter clinical trials in the near future and will be fundamental in establishing the viability of T cell GT for IEIs. Several exciting pre-clinical proof-of-concept studies have been published or are in development of gene edited T cell strategies and are detailed in this section. The T cell defects targeted for correction by gene editing are shown in Figure 2.

#### 1.5.1 CD40 Ligand Deficiency

CD40 ligand deficiency (also known as X-linked hyper-IgM syndrome) is an IEI that results from mutations in the CD40LG gene. CD40 ligand is expressed on the surface of activated T cells. CD40 is expressed on B cells thus, CD40 ligand is critical for T-B cell interactions including the induction of class switch recombination of the immunoglobulin heavy chain gene, antibody maturation, B cell proliferation, and the generation of long term memory responses (Hubbard et al., 2016; de la Morena et al., 2017; Kuo et al., 2018). Affected individuals suffer recurrent severe infections (particularly Pneumocystis jiroveci and Cryptosporidia species), autoimmune manifestations and have an increased risk of developing malignancies (Hubbard et al., 2016; Kuo et al., 2018). Classically, IgG, IgA, and IgE are absent whereas IgM levels are normal or elevated (Kuo et al., 2018). The disease results in premature mortality with patients surviving a median of 25 years after diagnosis with conservative management (de la Morena et al., 2017). AlloHSCT is curative however, high rates of GvHD and hepatic veno-occlusive disease have been observed and in a large cohort of patients there was no difference in overall survival between those treated with and without alloHSCT (de la Morena et al., 2017; Ferrua et al., 2019b).

Autologous GT would theoretically offer a curative therapy without many of the complications of alloHSCT. Due to the expression of CD40 ligand on T cells, the disease was considered as an early candidate for T cell GT. Early attempts, using retroviral vectors with constitutive promoters to correct murine models of CD40 ligand deficiency resulted in abnormal lymphoproliferation (Sacco et al., 2000). Successful transduction of CD4^+^ T cells was achieved with a self-inactivating lentivirus vector that used a stretch of the endogenous CD40 ligand promoter to drive gene expression. T cells taken from patients with CD40 ligand deficiency that were transduced with this vector exhibited and expression pattern close to normal physiology with kinetics between resting and activated states similar to WT T cells (Romero et al., 2011). Whilst this was an improvement over previous attempts with retroviruses, the lymphoproliferation observed in the murine model demonstrated the need for tight regulation of the CD40 LG gene.

Gene editing to achieve targeted gene correction with maintenance of the endogenous gene control machinery offers a clear advantage in CD40 ligand deficiency. Gene editing of human T cells for the correction of CD40 ligand deficiency was first achieved by Hubbard et al. (2016) who used TALENS to create a dsDNA break in the 5’ untranslated region (UTR) in the CD40LG gene. A recombinant AAV template was used to induce HDR and insert the CD40 ligand cDNA at the site of the double strand break. The authors demonstrated that following gene editing, CD40 ligand expression and CD40 binding were restored with physiological expression kinetics. Edited CD4^+^ T cells from patients with CD40 ligand deficiency were able to induce naïve B cell class switching at rates similar to those seen with healthy donor T cells. In comparison, unedited CD4^+^ T cells from patients were unable to induce B cells to undergo IgG class switch *in vitro*. Finally, the authors demonstrated that edited T cells had stable gene expression and were able to survive *in vivo* in a humanised immunodeficient mouse model (Hubbard et al., 2016).

Two years following the work of Hubbard et al. (2016), a different group used both TALEN and CRISPR/Cas9 with AAV templates to perform site-specific gene editing of HSCs for hyper IgM syndrome. Kuo et al. (2018) published editing efficiencies of up to 20% in HSCs [compared to 46% by Hubbard et al. (2016), in T cells] and argued that in comparison to T cell genome editing, a HSC strategy could provide permanent and robust immune reconstitution (Kuo et al., 2018).

A third group devised a universal editing strategy for CD40 ligand deficiency and compared T cell to HSC editing as potential therapeutic strategies. Vavassori et al. (2021), targeted the first intron of CD40LG using CRISPR/Cas9 and demonstrated that an artificial splice acceptor sequence could be used to achieve normal splicing of exon 1 onto the rest of the inserted cDNA. Editing efficiencies of up to 35% were achieved in patient CD4^+^ T cells but importantly showed that the CD62L+ CD45RA+, T stem cell memory subset (TSCM) was preserved in the edited T cell population (Vavassori et al., 2021). This subset has important clinical implications as this population has long-term multipotent and self-renewal capacity (Gattinoni et al., 2011; Oliveira et al., 2015).

Vavassori et al. (2021) added a C-terminal truncated NGFR sequence to the donor template to enable edited cells to be selected so that a cell product enriched for edited cells could be produced. Despite the increased length of this HDR donor, similar editing efficiencies were achieved in T cells as to cells edited with the shorter donor template (without the reporter). The authors then went on to edit HSCs with the same strategy and gene editing efficiencies of 30% were maintained in peripheral blood after engraftment in a humanised immunodeficient mouse model. Transient p53 inhibition by co-electroporation of an mRNA encoding for a dominant negative P53 truncated form was used to improve the degree and speed of engraftment of the edited HSCs (Vavassori et al., 2021).

Despite successfully editing both T cells and HSCs Vavassori et al. (2021), provided further data to support a T cell approach over HSCs for CD40 ligand deficiency. In addition to the data showing a high percentage of editing in the TSCM compartment, the authors showed that *ex vivo* culture (necessary for the production of an edited T cell product) did not negatively affect CD4^+^ T cell engraftment. In addition, comparison of T cell and HSC approaches were performed in adoptive transfer experiments of murine donor cells harvested from WT mice previously housed with CD40^−/−^ mice infected with *P. murina*, to simulate an autologous source of antigen primed cells (Vavassori et al., 2021). Negative control CD40^−/−^ mice developed interstitial pneumonia with a high burden of *P. murina* whereas mice who underwent adoptive transfer of WT T cells or HSCs showed very limited evidence of infection suggesting that a T cell or HSC approach would be successful in restoring humoral and macrophage-mediated responses. The transfer of T cells was equal if not more effective than HSC transplantation when the fraction of functional HSCs was limited to represent the levels achievable by HSC editing protocols. Given an autologous T cell GT approach has fewer safety concerns and would be easier to translate due to increased editing efficiencies compared to HSCs, Vavassori et al. (2021), positioned edited T cell GT ahead of edited HSC GT for CD40 ligand deficiency.

CD40 ligand deficiency is an excellent example of where T cell gene editing may offer a highly effective therapeutic approach. Whilst, human clinical trials will be needed to assess the efficacy of T cell gene edited GT, the proof-of-concept studies described suggest that a T cell strategy would be as effective as a HSC strategy in terms of clinical efficacy. Promising data on the high degree of editing of the TSCM compartment suggests that a T cell approach may be able to provide durable clinical responses. CD40 ligand deficiency is the IEI for which a gene edited T cell therapy is at the most advanced stage of development and it is expected that one or more of these approaches will enter clinical trials in humans in the near future.

#### 1.5.2 Immune Dysregulation Polyendocrinopathy Enteropathy X-Linked Syndrome

Proof-of-concept GT approaches using viral vector gene addition technology for IPEX syndrome were described in an earlier section of this review. Viral vector gene addition techniques have several limitations in IPEX syndrome. Firstly, transduction of IPEX patient conventional T cells with a lentiviral vector (expressing FOXP3 under a constitutive EF1a promoter) converted them to potent Treg like suppressor cells but this approach does not address the defect in other cell types such as T effector cells themselves which contribute to the pathophysiology of IPEX syndrome (Goodwin et al., 2020). Proof-of-concept GT approaches using viral vector gene addition technology for IPEX syndrome were described in an earlier section of this review. Viral vector gene addition techniques have several limitations in IPEX syndrome. Firstly, transduction of IPEX patient conventional T cells with a lentiviral vector (expressing FOXP3 under a constitutive EF1a promoter) converted them to potent Treg like suppressor cells but this approach does not address the defect in other cell types such as T effector cells themselves which contribute to the pathophysiology of IPEX syndrome (Goodwin et al., 2020). An additional limitation of viral gene-addition approaches in IPEX syndrome is that they cannot be applied to HSCs due to the toxicity of FOXP3 on HSCs in terms of stem cell proliferation and differentiation. Whilst a degree of long-term persistence will likely be seen with a T cell approach, due to the as yet unproven nature of T cell GT for IEIs, the parallel development of an HSC strategy is warranted (Goodwin et al., 2020; Melenhorst et al., 2022).

A proof-of-concept gene editing approach for IPEX syndrome has been published. Goodwin et al. (2020), designed a gRNA that crossed the start codon of *FOXP3*. They then designed an AAV6 HDR donor that would insert a codon divergent *FOXP3* cDNA template followed by a polyadenylation signal, a constitutive promoter, and a truncated nerve growth factor receptor (tNGFR) reporter gene. The NGFR reporter was placed under the control of a constitutive promoter so that expression of this gene was independent of FOXP3, thus enabling edited HSCs to be selected based on NGFR expression (FOXP3 is not expressed in HSCs).

The authors then used this approach to edit both human T cells and HSCs. Rates of HDR mediated editing were 24% ± 10% in CD4^+^ T cells and 23% ± 5% in HSCs. In the edited T cells, FOXP3 expression persisted and Tregs maintained their immunophenotype and function (assessed as their ability to suppress the proliferation of activated T effector cells). The authors then edited T cells from six patients with IPEX syndrome (five different mutations). Gene editing efficiency in patient Tregs was comparable to healthy donor T cells and restored FOXP3 expression. Restoration of FOXP3 by gene editing, normalised proliferation rates to those observed in healthy donor T effector cells (Goodwin et al., 2020). These data provide promising pre-clinical proof-of-concept of an autologous gene editing therapeutic approach for IPEX syndrome. Although the authors demonstrate successful T cell and HSC editing, it is the opinion of these authors that a T cell approach may offer significant advantages over HSC editing, notably easier clinical translation, lower safety concerns and comparable clinical benefits (Vavassori et al., 2021). Although, persistence of gene edited T cells will need to be assessed in human clinical trials, similar to the work on CD40 ligand deficiency by Vavassori et al. (2021), we would position a T cell approach ahead of HSCs for initial assessment.

#### 1.5.3 X-Linked Lymphoproliferative Disease

As discussed previously, an autologous T cell therapy could offer an attractive intervention to treat X-linked lymphoproliferative disease, and it has been shown that a lentiviral gene delivery approach is able to restore the functional defects seen in XLP patient T cells (Panchal et al., 2018b) and a future clinical trial will investigate the durability of this approach In addition to this work, a gene editing strategy has been investigated which has the potential to harness regulatory elements in the SH2D1A promoter which may replicate more faithfully the expression profile; SAP has a regulated profile, with expression restricted in Tregs, and alteration in expression after TCR engagement or with memory phenotype (Mehrle et al., 2005; Hale et al., 2013).

Houghton et al. (2022) investigated the use of three classes of site-specific nuclease (TALEN, CRISPR-Cas9, and CRISPR-Cas12a), to target sites early in first exon of the SH2D1A gene. Nucleofection of TALEN mRNA and CRISPR-Cas9 RNPs were particularly effective, creating >90% SAP protein knock down in healthy cells, while Cas12a RNPs gave >50%. AAV6-based HDR donors co-expressing SAP and a GFP reporter (separated by a P2A self-cleaving peptide) were able to drive HDR-mediated gene insertion rates of over 45% in bulk patient T cells, with resulting SAP protein expression closely matching that of unedited healthy controls (Houghton et al., 2022). Importantly in the XLP setting, rates of editing were equivalent in CD4 and CD8 compartments. In an additional optimisation, they found that a serum-free AAV6 transduction protocol allowed for reduction in AAV dose without loss in HDR efficiency, which could save production costs and improve patient access in the clinical trial setting. GE XLP patient T cells demonstrated functional restoration in three *in vitro* assays, including restoration of sensitivity to restimulation induced cell death (RICD), restored T follicular helper cell function and restored cytotoxicity against EBV-infected B cell targets.

#### 1.5.4 Cytotoxic T-Lymphocyte Antigen 4 Insufficiency

CTLA4 insufficiency is an IEI with a severe clinical phenotype that results from heterozygous germline mutations in CTLA4. CTLA4 insufficiency is a relatively new diagnosis, first described in 2014 (Kuehn et al., 2014; Schwab et al., 2018; Egg et al., 2022). Many patients with *CTLA4* gene mutations had previously been diagnosed with common variable immunodeficiency (CVID) and/or an autoimmune syndrome, however as availability of genetic diagnosis and awareness of the condition has improved the number of recognised cases has increased dramatically (Egg et al., 2022). It is inherited in an autosomal dominant fashion with incomplete penetrance. Common clinical manifestations include, hypogammaglobulinaemia, lymphoproliferation, autoimmune cytopenia as well as respiratory, gastrointestinal, and neurological presentations due to lymphoid infiltration (Schubert et al., 2014; Zeissig et al., 2015; Schwab et al., 2018). The median age of onset of symptoms in CTLA4 deficiency in the largest case series was 11 years old, however, this was highly variable and ranged from 1 to 59 years. Management is challenging and whilst the CTLA4 fusion protein mimetics (abatacept and belatacept) can result in clinical improvement, concomitant systemic immunosuppression is usually required to control autoimmunity (Lo et al., 2015; Verma et al., 2017; Schwab et al., 2018). AllloHSCT is therefore currently the only curative treatment however, it carries high risk of mortality as well as morbidity from graft failure, graft rejection and GVHD (Slatter et al., 2016; Schwab et al., 2018).

CTLA4 (CD152) is a critical negative immune regulator, expressed constitutively on Tregs and on conventional T cells upon activation (Khailaie et al., 2018; Rowshanravan et al., 2018). CTLA4 competes with CD28, for the shared ligands CD80 and CD86, expressed on antigen presenting cells (APCs). CTLA4 binds its ligands and then removes them from APCs by the process of transendocytosis (TE) thereby depleting the same ligands required for CD28 co-stimulation, causing immunosupression (Qureshi et al., 2011; Rowshanravan et al., 2018; Schwab et al., 2018). Due to expression of CTLA4 on T cells and particularly Tregs, it is another IEI for which a T cell autologous product may resolve the clinical phenotype. As tightly regulated gene expression is required, a specific gene editing approach may be more appropriate facilitating physiological, dynamic, cell-specific protein expression. Several groups are working on T cell gene editing [Fox et al. (2018), American Society for Gene and Cell Therapy annual congress abstracts 2022 abstract 484, Marson group pre-print (Shy et al., 2021)] for CTLA4 insufficiency and peer-reviewed results are expected soon.

### 1.6 *In Vivo* Gene Editing of T Cells

Whilst *ex vivo* gene therapy has great potential, it is limited to cells which can be easily accessed and manipulated *ex vivo*. The procedure requires a sophisticated infrastructure, that is only available in advanced health care systems and the need for pre-conditioning with lymphodepletion has side effects. The ability to correct genetic defects *in vivo* using gene editing would be a huge advance. The difficulty of *in vivo* editing is targeting a specific tissue and avoiding off-target unwanted edits.

Successful *in vivo* gene editing has recently been achieved in humans for transthyretin amyloidosis (Gillmore et al., 2021). This innovative strategy delivered a single gRNA targeting the transthyretin gene (*TTR*) and the Cas9 protein using a lipid nanoparticle (LNP). The LNP was optimised for delivery to hepatocytes (the site of TTR manufacture) as plasma apolipoprotein E opsonises the LNP surface so that it is actively endocytosed by hepatocytes (Akinc et al., 2019). The gene editing results in indels in the *TTR* gene preventing production of functional TTR protein. Clinical results were dramatic, with durable knock down demonstrated after a single dose and significant decreases in serum TTR protein concentrations (Gillmore et al., 2021).

Emerging data suggests that lentivirus-based virus like particles (VLPs) may be able to perform *in vivo* gene editing of specific T cell populations (Hamilton et al., 2021). VLPs are assemblies of viral proteins that are able to infect cells but lack the viral genetic material avoiding the risks associated with viral integration and prolonged expression of the gene editing machinery (Banskota et al., 2022). The authors of a recently published study developed a one-step strategy that uses VLPs to introduce Cas9 RNPs and a HDR template into primary human T cells without electroporation (Hamilton et al., 2021). The authors used this approach to introduce a CAR construct whilst simultaneously knocking out genetic targets relevant to allogeneic CAR-T cell production. Clinically relevant editing frequencies were obtained with this approach and the authors demonstrated that the CD4 fraction could be targeted alone by modifying the viral envelope (Hamilton et al., 2021). More recently, VLPs have been applied *in vivo* and have successfully been used to perform base editing in the eye, brain and liver (Banskota et al., 2022). The same group demonstrated that VLPs can successfully perform base editing in mouse and human T cells *ex vivo* (Banskota et al., 2022). Together, these data highlight the potential of using VLPs to perform *in vivo* gene correction. Whilst it will likely be several years before *in vivo* gene editing of T cells enters the clinic, these recent developments suggest that it may be possible to selectively edit the T cell compartment without the need for *ex vivo* manipulation. Should successful *in vivo* editing be achieved this would make a T cell approach even more attractive to correct IEIs than editing less accessible HSCs.

## 2 Conclusion

The rapid progress in the field of gene therapy, and specifically gene editing, has enabled the development of novel approaches to treat IEI. Based on past and current experience of adoptive transfer of gene modified T cells for infective, immunological and malignant conditions, proof of concept studies for T cell gene therapy in IEI are moving toward clinical trial including, some using a gene editing platform to correct patient cells. The manufacture processes, cryopreservation techniques and clinical treatment protocols are being established and optimised for gene edited T cell therapies in the context of haematological malignancies and this experience will provide knowledge and infrastructure support which is readily transferrable to monogenic diseases. Gene edited T cell therapy has the potential to offer a safe and effective therapeutic option for a wide range of IEI and immune dysregulatory disorders; *in vivo* persistence of gene modified T cells and the durability of treatment effect will be borne out in clinical trials. Advances in *in vivo* gene editing may enable correction of some of these diseases without the need for *ex vivo* manipulation of T cells, further improving the attractiveness of this approach.
